# Case Report: long-term misdiagnosis and follow-up of a patient with *HNF4A*-MODY carrying a new *de novo* mutation

**DOI:** 10.3389/fendo.2025.1509135

**Published:** 2025-06-16

**Authors:** Jing Luo, Ai Li, Xiaoli Wang

**Affiliations:** ^1^ Department of Endocrinology and Metabolism, Institute of Endocrinology, NHC Key Laboratory of Diagnosis and Treatment of Thyroid Diseases, The First Hospital of China Medical University, Shenyang, China; ^2^ Department of Endocrinology and Metabolism, Tieling Central Hospital, Tieling, China

**Keywords:** HNF4A, maturity-onset diabetes of the young, mutation, case report, GLP1RA

## Abstract

**Background:**

*HNF4A*-MODY constitutes 5%–10% of MODY cases; however, treatment options remain unclearly recommended, and long-term follow-up of patients with *HNF4A*-MODY is lacking due to limited research. Here, we report a case carrying a new *de novo* variant of *HNF4A*. The patient had been using insulin for up to 25 years before genetic diagnosis.

**Case description:**

A 38-year-old man sought consultation due to an increased daily insulin requirement and inadequate glycemic control. At the age of 13, the patent’s parents discovered that he had significantly elevated fasting blood glucose levels accompanied by weight loss. He was subsequently diagnosed with type 1 diabetes and began insulin therapy. At a routine follow-up at age 21, another physician observed that his pancreatic islet function remained preserved, with negative results for diabetes-related antibodies. Consequently, his diagnosis was revised to type 2 diabetes, and the antihyperglycemic therapy was added in metformin and acarbose. Before the current consultation, the patient’s insulin dosage had gradually increased to 80 units per day; however, glycemic control remained unsatisfactory. Whole exome sequencing identified a heterozygous variant, c.272G > A (p.R91H), in exon 3 of the *HNF4A* gene (NM_175914.5) in the patient. The patient’s treatment regimen was modified to include metformin at a dosage of 1.0 g twice daily, semaglutide at 0.5 mg once weekly, and insulin glargine was gradually discontinued. The patient achieved adequate glycemic control during follow-up.

**Conclusion:**

This case emphasizes that spontaneous *HNF4A*-MODY is prone to misdiagnosis and the prolonged rate of pancreatic function decline in *HNF4A*-MODY. Glycemic control and complication progression could be acceptable in *HNF4A*-MODY cases treated with long-time insulin, but risks of hypoglycemic events, obesity, and atherosclerosis remain. Switching to GLP1RA treatment in *HNF4A*-MODY still yields a good effect after a prolonged disease course.

## 1 Introduction

Hepatocyte Nuclear Factor 4 Alpha (*HNF4A*)-related maturity-onset diabetes of the young (MODY) constitutes approximately 5% to 10% of all MODY cases (1); however, the literature documents only around 20 instances of *HNF4A*-MODY in China. *HNF4A*-MODY has demonstrated responsiveness to sulfonylureas (2), and GLP-1 receptor agonists (GLP-1RAs) have proven effective in achieving satisfactory glycemic control in select cases (3, 4). Nevertheless, treatment modalities remain insufficiently characterized due to insufficient research (5). Limited studies have explored the long-term management of patients diagnosed with *HNF4A*-MODY.

Here, we report a case from China involving a new *de novo* variant of *HNF4A*. The patient had been using insulin for up to 25 years before receiving a genetic diagnosis.

## 2 Case report

**Figure 1:**
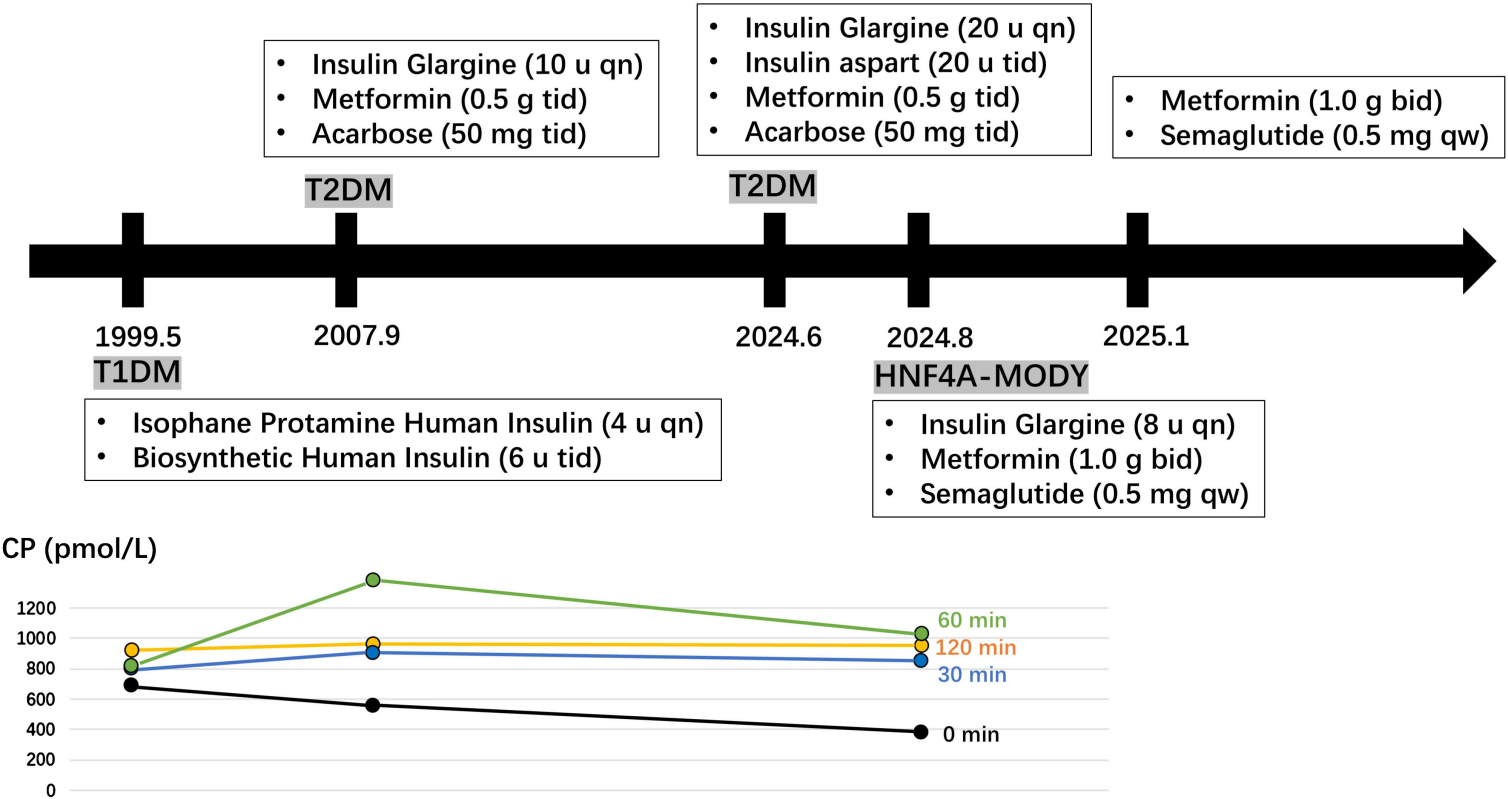
A historical timeline of the patient. Fasting and postprandial C-peptide (CP) levels were measured at the ages of 13, 21, and 38 years.

At the age of 13, the patent’s parents discovered that he had significantly elevated fasting blood glucose levels (320.4 mg/dL) accompanied by weight loss. He was subsequently diagnosed with type 1 diabetes and began insulin therapy. During this period, he experienced multiple hypoglycemic episodes. At a routine follow-up at age 21, another physician observed that his pancreatic islet function remained preserved, with negative results for diabetes-related antibodies. Consequently, his diagnosis was revised to type 2 diabetes, and the antihyperglycemic therapy was added in metformin and acarbose. At the age of 38, the patient sought consultation due to an increased daily insulin requirement and inadequate glycemic control. Before the current consultation, the patient’s insulin dosage had gradually increased to 80 units per day; however, glycemic control remained unsatisfactory, as indicated by an HbA1c level of 9.6% (**Figure 1**).

The patient was born with a birth weight of 4,300 g; however, there was no relevant medical record indicating neonatal hypoglycemia. Additionally, the family reported no history of diabetes. At 21 years of age, the patient had a body mass index (BMI) of 24.5 kg/m²; at 38 years of age, the BMI increased to 28.4 kg/m². His blood pressure was recorded at 145/90 mmHg. Laboratory tests for serum lipid levels, uric acid, diabetic autoimmune antibodies, urinary microalbumin creatinine ratio, and thyroid function, including antibodies, were all within normal ranges. Fasting and postprandial C-peptide levels were measured at ages 13, 21, and 38 years (**Figure 1**). A carotid ultrasound indicated the presence of atherosclerosis, and a fundoscopic examination revealed scattered macular hemorrhages in both eyes.

### 2.1 Genetic testing and counseling

**Figure 2:**
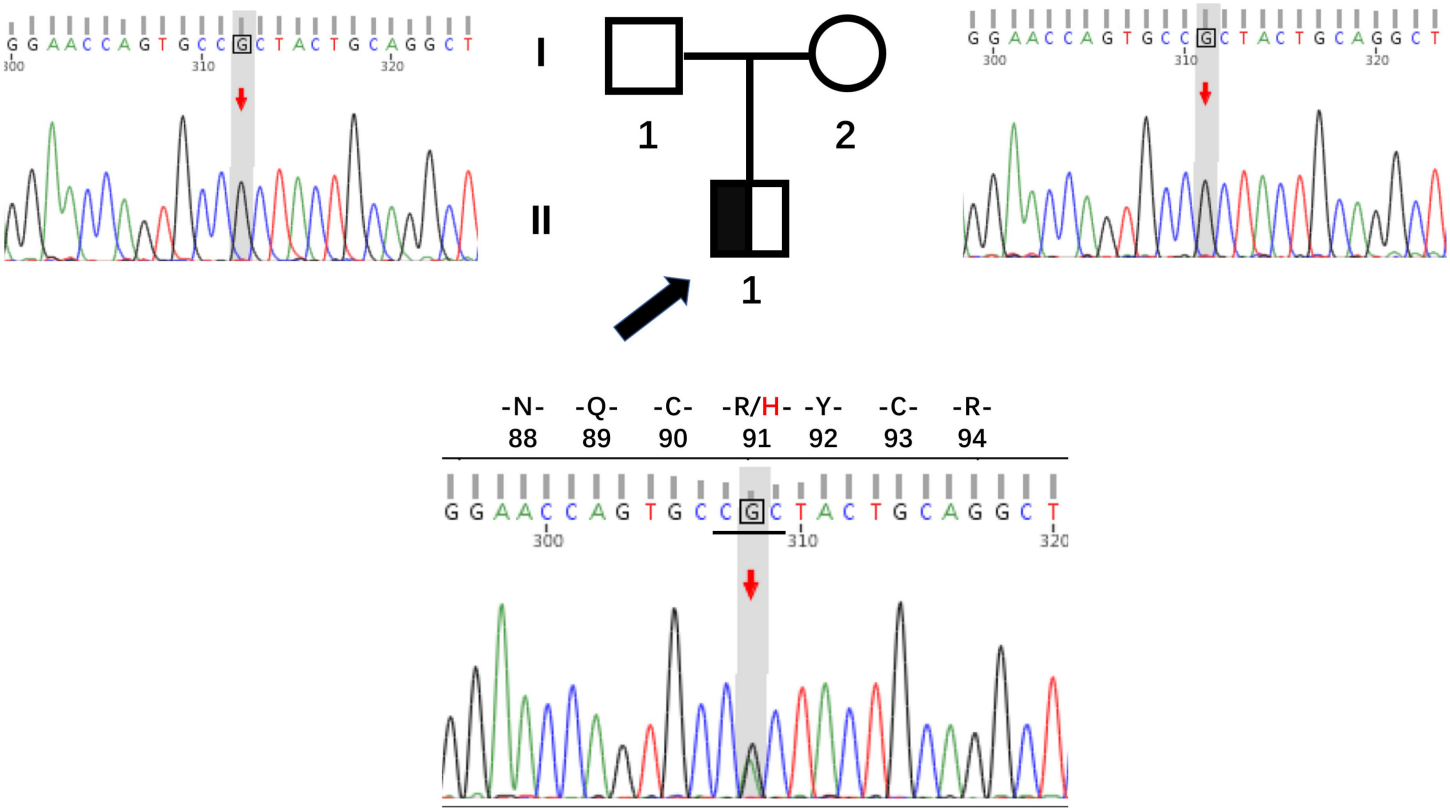
Pedigrees and sequencing chromatograms for the family with the *HNF4A* mutation R91H. The proband is indicated by a black arrow, with males represented by squares and females by circles.

Whole exome sequencing was performed to identify a genetic etiology, given that the patient presented with early-onset diabetes, reserved pancreatic function, and negative diabetes-related antibodies. As anticipated, we identified a novel heterozygous variant, c.272G > A (p.R91H), in exon 3 of the *HNF4A* gene (NM_175914.5) in the patient. Sanger sequencing confirmed the presence of this variant in the patient but not in his parents (**Figure 2**). Recent versions of genomic databases did not report this missense variant; according to the ACMG/AMP guidelines, it is classified as likely pathogenic (PM1, PM2, PP1, and PP3). Thus, the diagnosis for this patient is classified as *HNF4A*-MODY.

This *de novo* mutation might originate from early embryonic mutations or parental germline mosaicism. Even though the tests on the parents are negative, there is still an extremely slight risk of recurrence because of the potential mosaicism. We suggest that the parents consider undergoing high-sensitivity testing to rule out low-level mosaicism. However, due to their advanced age, the parents have no plans for another pregnancy and thus have no intention of undergoing further examinations. For the patient, there is a 50% chance that their offspring will develop the disease. If the patient has plans for childbearing, preimplantation genetic testing or prenatal diagnosis options can be considered.

### 2.2 Treatment and Follow-up

The patient’s treatment regimen was modified to include metformin at a dosage of 1.0 g twice daily, semaglutide at 0.5 mg once weekly, and insulin glargine gradually reduced to 8 units once daily. After six months, the patient achieved adequate glycemic control, as indicated by hemoglobin A1c levels of 7.1%—7.3%. The patient’s achieved a weight loss of 4.2 kilograms, and insulin has been discontinued at present.

The patient expresses a high level of satisfaction with the current treatment regimen and is willing to comply with scheduled follow-up appointments.

## 3 Discussion

**Figure 3:**
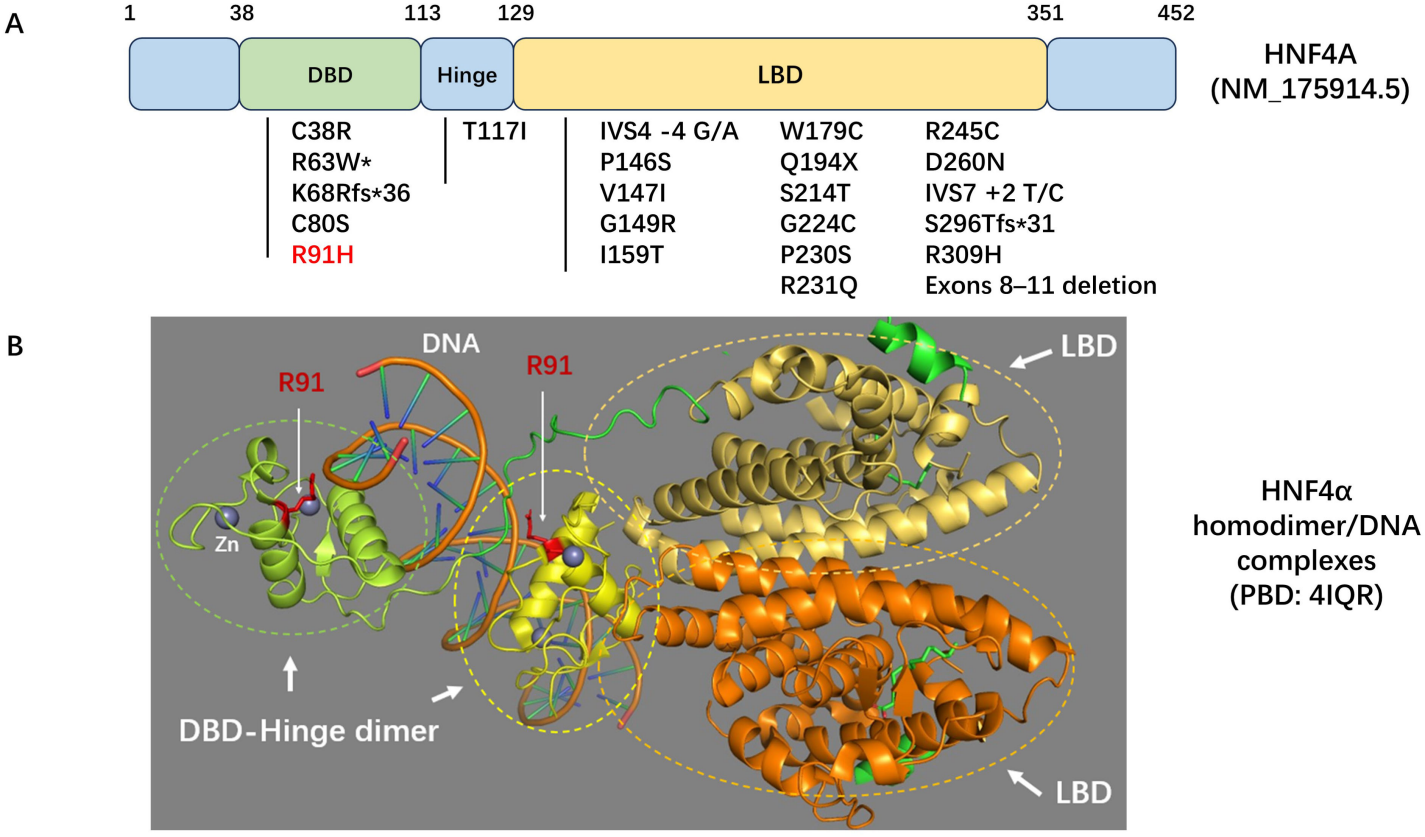
**(A)** Schematic diagram of the *HNF4A* gene. The locations of variants identified in Chinese patients with *HNF4A*-MODY or congenital hyperinsulinemia are indicated. *This condition has also been reported as *HNF4A*-related Fanconi syndrome. **(B)** Homodimer/DNA complex model of HNF4α. The ligand binding domains (LBD) are depicted in yellow-orange and orange, respectively. The DNA binding domain (DBD)-hinge dimers are illustrated in green and yellow. Residue R91, a component of the DBD-hinge dimer interface in HNF4α, is highlighted in red. Zinc (Zn) is represented in grey. The Protein Data Bank (PDB) ID code for this model is 4IQR.

The *HNF4A* gene encodes HNF4α, a member of the nuclear receptor superfamily of ligand-dependent transcription factors, which play a crucial role in regulating pancreatic insulin secretion (2). *HNF4A*-MODY accounts for approximately 5% to 10% of all MODY cases; however, it is infrequently observed in the Chinese population (6, 7). We reviewed the literature and identified 23 mutations in the *HNF4A* gene among Chinese patients (**Figure 3**). The majority of these mutations are localized within the DNA binding domain (DBD) and ligand binding domain (LBD) regions. Notably, the R91H mutation in this patient is located in the highly conserved DBD region of HNF4α (8) (**Figure 3**).

This case emphasizes that spontaneous *HNF4A*-MODY is highly susceptible to misdiagnosis as either type 1 or type 2 diabetes mellitus. Currently, there are recommended tools for screening the clinical risk for MODY, such as the AACM strategy, which includes the age of onset, autoantibody to islet antigen, C-peptide and metabolic syndrome (9), and the MODY probability calculator (10). However, these tools have demonstrated inadequate efficacy in distinguishing MODY cases (11). Moreover, epidemiological research has indicated that *de novo* mutations in the MODY genes may occur more frequently than previously estimated (12). In individuals suspected of having MODY, characterized by clinical indicators such as macrosomia, early onset, absence of obesity, non-insulin dependence, and negative diabetic autoimmune antibodies—regardless of family history—a genetic assessment should be considered.

There is a paucity of research investigating the long-term follow-up and management strategies for patients diagnosed with *HNF4A*-MODY. Prolonged follow-up of this patient indicated a gradual decline in pancreatic function associated with *HNF4A*-MODY. Sulfonylureas are recognized as the standard first-line therapy for *HNF4A*-MODY (2). Due to the patient’s obesity, we kept metformin in the treatment regimen. Additionally, we gradually reduced the insulin dosage and avoided sulfonylurea medications that stimulate insulin secretion, in order to avoid the risk of atherosclerotic cardiovascular disease (ASCVD) associated with weight gain. It is reasonable to hypothesize that patients with *HNF4A*-MODY may respond positively to meglitinides and GLP-1RAs; however, there is currently no empirical evidence to support this hypothesis. While glycemic control and the progression of complications can be effectively managed in *HNF4A*-MODY patients receiving long-term insulin therapy, the potential risks of hypoglycemic episodes, obesity, and atherosclerosis remain significant concerns. Furthermore, the shift to GLP-1RA treatment in individuals with *HNF4A*-MODY continues to exhibit beneficial outcomes, even after an extended period of disease progression.

In summary, we have identified a novel *de novo* heterozygous missense mutation in the *HNF4A* gene in a Chinese patient diagnosed with MODY. This finding contributes to the existing mutation spectrum associated with *HNF4A*. This case highlights the susceptibility of spontaneous *HNF4A*-MODY to misdiagnosis, as well as the prolonged decline in pancreatic function characteristic of this condition. Notably, transitioning to GLP-1RA treatment in individuals with *HNF4A*-MODY continues to demonstrate positive outcomes, even after an extended disease duration.

## Data Availability

The datasets presented in this study can be found in online repositories. The names of the repository/repositories and accession number(s) can be found here: https://doi.org/10.6084/m9.figshare.29236490.
